# Prognostic Value of Circulating Tumor DNA (ctDNA) in Oncogene-Driven NSCLC: Current Knowledge and Future Perspectives

**DOI:** 10.3390/cancers14194954

**Published:** 2022-10-10

**Authors:** Eleni Zografos, Foteinos-Ioannis Dimitrakopoulos, Angelos Koutras

**Affiliations:** 1 Division of Oncology, University Hospital of Patras, University of Patras, 26504 Patras, Greece; 2 Molecular Oncology Laboratory, Division of Oncology, Department of Medicine, University of Patras, 26504 Patras, Greece

**Keywords:** ctDNA, liquid biopsy, minimal residual disease (MRD), NSCLC, driver mutations

## Abstract

**Simple Summary:**

Personalized medicine has significantly changed the clinical outcome of oncogene-driven non-small cell lung cancer (NSCLC) due to the efficacy of molecular targeted therapies. Despite the advances in the management of this group of patients, the need for powerful biomarkers with the potential for a real-time assessment of the tumor genomic profile as well as for detecting and monitoring minimal residual disease (MRD) remains unmet. The aim of this article is to present the current knowledge and the future perspectives regarding the prognostic value of ctDNA in NSCLC, focusing on the most common druggable driver mutations, including those in epidermal growth factor receptor (*EGFR*), anaplastic lymphoma kinase (*ALK*), c-ros oncogene 1 (*ROS1*), rearranged during transfection (*RET*), kirsten rat sarcoma virus (*KRAS*), B-Raf proto-oncogene (*BRAF*), and mesenchymal epithelial transition factor receptor (*MET*) genes.

**Abstract:**

As we enter an unprecedented era of personalized medicine, molecular targeted therapies have the potential to induce improved survival outcome in patients with non-small cell lung cancer (NSCLC). However, a significant percentage of oncogene-driven NSCLC patients will relapse even after definitive treatment, whereas chronic and durable response to targeted therapies is a less common event in advanced-stage lung cancer. This phenomenon could be attributed to minimal residual disease (MRD), defined as a population of disseminated tumor cells that survive during the course or after treatment, eventually leading to recurrence and limiting patient survival. Circulating tumor DNA (ctDNA) is a powerful biomarker for MRD detection and monitoring and is a non-invasive approach of treating cancer, and especially NSCLC, based on a real-time assessment of the tumor genomic landscape. In this review, we present the key findings of studies that have used ctDNA with regard to its prognostic value and in respect to the most common druggable driver mutations of genes in NSCLC, such as epidermal growth factor receptor (*EGFR*), anaplastic lymphoma kinase (*ALK*), c-ros oncogene 1 (*ROS1*), rearranged during transfection (*RET*), Kirsten rat sarcoma virus (*KRAS*), B-Raf proto-oncogene (*BRAF*), and mesenchymal epithelial transition factor receptor (*MET*).

## 1. Monitoring of Minimal Residual Disease Using ctDNA

Precision medicine is rapidly evolving as an integral part of modern oncology, steering the field towards tailoring of therapeutic strategies based on the unique molecular features, tumor microenvironment, individual gene variability, environmental factors, and lifestyle [1]. In addition, so far, insurmountable obstacles in cancer research, such as drug resistance, genomic heterogeneity of tumors, and inadequate means for monitoring of tumor recurrence and treatment response, are expected to be surpassed by advances in precision medicine, finally improving the survival outcome of cancer patients [2]. 

During the last decade, precision medicine has started to transform the treatment landscape of lung cancer, which remains the leading cause of cancer-related deaths in both men and women worldwide, with an estimated 1.8 million deaths in 2020 [3]. The vast majority of patients with lung cancer fall under the broad histologic category of non-small cell lung cancer (NSCLC), which constitutes 85% of all cases. Adenocarcinomas and squamous cell carcinomas are the two most common histological subtypes of NSCLC [4]. Beyond histology, better understanding of the molecular background across all lung cancer types, along with the emerging importance of genetic testing, largely owing to the identification of targetable molecular abnormalities, have revolutionized the way we treat patients with NSCLC [5].

Surgery remains the cornerstone of early-stage NSCLC treatment, while adjuvant or neoadjuvant therapies are used in order to reduce recurrence rate [6,7]. Unfortunately, 30–50% of NSCLC patients will relapse even after undergoing a R0 tumor resection [8]. These high relapse rates suggest that a considerable fraction of these patients with theoretically successful initial treatment, most likely, suffer from micrometastatic disease at the time of surgery, which is clinically undetected and persists even after resection and adjuvant therapy, eventually acting as a latent source of local or distant recurrence [9].

In this setting, the concept of minimal residual disease (MRD) has been suggested to describe the small number of remaining cancer cells during the course or after the completion of treatment. These early disseminated cells, under the influence of specific signals originating either from the secondary organ’s microenvironment or from pre-encoded dormancy signatures initiated in the primary site by hypoxia, enter a dormant state that corresponds to cell-cycle arrest [10,11]. Dormancy is a process that encompasses cancer cell quiescence, angiogenic dormancy, where a balance between proliferating cells and those that perish due to insufficient vascularization keeps the tumor mass constant, and immune-mediated dormancy in which the tumor mass remains steady via persistent cytotoxic activity [12]. Residual disseminated tumor cells evade therapy until suddenly reawakened to initiate proliferation into clinically detectable macrometastases [13]. Verifying the presence of MRD acts as a predictive indicator for disease recurrence and overall survival in a similar manner to lymph-node metastasis, which serves as a marker of systemic disease [14]. 

However, the main challenge lies in MRD detection and monitoring, especially in solid tumor patients due to the technically difficult isolation of circulating tumor cells (CTCs) or factors that cancer cells extrude into the bloodstream, such as circulating tumor DNA (ctDNA). In particular, apoptotic and necrotic tumor cells release their fragmented DNA into the bloodstream, creating a new pool of genetic material, which can be used for further disease exploration in clinical settings [15]. The foremost advantage of ctDNA is that it reflects more accurately cancer spatial and temporal heterogeneity and, thus, allows tracking of the metastatic burden [16]. Furthermore, ctDNA profiling allows us to monitor the subclonal origin of cancer metastasis [17,18] and has emerged as a promising blood-based biomarker with the dynamics to advance the current understanding of metastasis [19]. Based on the growing volume of data, both the US Food and Drug Administration (FDA) and European Medicines Agency (EMA) approved in 2016 the first ctDNA-based “liquid biopsy” for the identification and quantification of somatic epidermal growth factor receptor (*EGFR*) mutations that allows us to pinpoint patients with NSCLC who will benefit from targeted therapy, specifically in cases where we are not able to obtain a tissue biopsy [20,21]. 

The unavailability of tissue samples for molecular profiling is a common problem in daily clinical practice. Tumor sampling in its entirety is invasive and challenging, while repeated tissue biopsies of suspicious primary or metastatic lesions may be difficult to be scheduled on time and lead to potential procedure-related complications [22]. In parallel, tumor dynamics or sensitivity to treatment, which reflect plasticity and heterogeneity of cancer, are not properly detected using conventional approaches. In addition, regarding follow-up of NSCLC patients, the most crucial disadvantage of radiological assessment is that imaging is not able to detect minimal residual disease, but only space-occupying lesions. Furthermore, imaging modalities harbor a relatively small but existing risk of radiation over-exposure [23]. On the other hand, diagnosing and screening through non-invasive methods represents an important paradigm shift in precision medicine. With the development of sensitive techniques that can detect genetic or epigenetic alterations, we can determine the heterogeneous tumor landscape and even capture its dynamics over time, using a simple blood sample. In this context, liquid biopsy approaches enable MRD monitoring, thereby contributing towards the identification of those who face a high risk of disease relapse following initial therapy [24].

**Figure 1:**
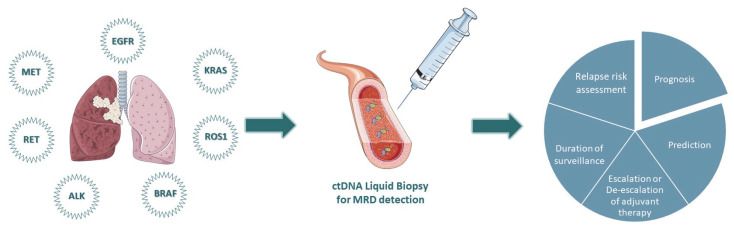
Rationale of personalized ctDNA-based detection of minimal residual disease in patients with oncogenic-driven NSCLC.

In this review study, we focus on the available data regarding the prognostic value of ctDNA in patients with NSCLC who carry druggable genetic driver mutations in significant genes, such as epidermal growth factor receptor (*EGFR*), anaplastic lymphoma kinase (*ALK*), c-ros oncogene 1 (*ROS1*), rearranged during transfection (*RET*), Kirsten rat sarcoma virus (*KRAS*), B-Raf proto-oncogene (*BRAF*), and mesenchymal–epithelial transition (*MET*). Due to the immense literature on the predictive value of ctDNA in NSCLC, the scope of our study was limited to the prognostic significance of ctDNA analysis (Figure 1).

## 2. Minimal Residual Disease, ctDNA, and Oncogene-Addicted NSCLC

NSCLC is a heterogeneous entity that encompasses several druggable mutations, each of which needs distinct treatment management with particular clinical outcome [25]. Oncogene-addicted NSCLC is mainly characterized by a somatic mutation detected in a specific oncogene that drives tumor proliferation and is predictive of drug activity [26]. In this setting, the latest guidelines recommend up-front testing for *EGFR* activating mutations, *ALK* and *ROS1* fusions, and activating exon 15 V600E *BRAF* point mutations [7]. Furthermore, other therapeutically targetable driver and resistance alterations include *MET* amplifications and *MET* exon 14 skipping variants, as well as *RET* and *NTRK* rearrangements. Numerous ongoing clinical trials are currently testing therapies that target *HER2* activating mutations, although data are still accumulating [27]. Activating variations in the *KRAS* gene should also be evaluated at NSCLC diagnosis since their presence excludes other targetable driver mutations and are prognostic of poor survival [18,28]. In addition, *KRAS* p.G12C has been documented as a sensitizing mutation associated with responsiveness to the oral RAS-GTPase inhibitor sotorasib, offering an additional therapeutic option for the targeted treatment of NSCLC [29]. Similar to sotorasib, adagrasib is the second selective *KRAS* G12C inhibitor to have been shown with clinical efficacy against patients with previously treated *KRAS* G12C-mutated NSCLC, according to the results of the KRYSTAL-1 multi-cohort phase II study (NCT03785249) [30].

Although the aforementioned genomic targets in NSCLC can be effectively identified through sensitive and comprehensive sequencing of tumor specimens, they can also be assessed using plasma samples and ctDNA. In fact, the concordance of driver mutation detection between tissue and blood specimens in patients with NSCLC has been well established in various publications [31]. For example, in a prospective study which compared tissue with ctDNA genotyping in newly diagnosed stage IIIB–IV NSCLC, clinical sensitivity of ctDNA for the detection of actionable genomic alterations was greater than 98.2% with a significantly shorter turnaround time [32]. 

Furthermore, ctDNA evaluation can also be used as a predictor of relapse risk in NSCLC patients [22]. It is well known that the benefit of adjuvant chemotherapy in early-stage NSCLC is modest, translating into an absolute 5-year survival advantage of approximately 5% [33]. These data suggest that there is lack of biomarkers that can predict innate tumor behavior and can identify high-risk patients. The possibility of better defining the small proportion of patients most likely to derive survival benefit from adjuvant therapy and sparing patients who do not need complementary treatments is, therefore, particularly important. MRD is under assessment in several ongoing trials in which NSCLC patients, treated with curative intent, are recruited, including the phase III MERMAID-1 and 2 trials. The aim is to identify and monitor residual disease after primary stage II–III NSCLC surgical resection, improving outcomes in the adjuvant setting. Eventually, this approach might turn the therapeutic focus exclusively towards MRD-positive patients destined to recur; following genomic characterization, these patients would be the only ones to receive targeted treatment against the isolated tumor subclone. Looking back, the TRACERx study was one of the first, which investigated the ability of ctDNA to predict postoperatively NSCLC relapse, by performing multiplex-PCR next generation sequencing (NGS) of pre- and post-surgical ctDNA [17]. Specifically, Abbosh et al. analyzed resection specimens to develop a patient-specific panel of single nucleotide variants present in the primary lung tumor. Based on this mutational panel, they showed that persistent detection of ctDNA after surgery predicted relapse in 93% of patients with an average time gap of 70 days prior to radiologic diagnosis of cancer recurrence. Alternatively, Chaudhuri et al. adopted a non-targeted deep sequencing approach that does not require detailed information for each patient and demonstrated that in 94% of patients with localized lung cancer, postoperative ctDNA detection was correlated with subsequent relapse [34]. Interestingly, ctDNA detection preceded radiological progression in 72% of patients, with a median lead interval of 5.2 months. Furthermore, patients with detectable ctDNA on a blood sample collected less than four months after surgery had a significantly worse outcome, in terms of relapse-free survival and overall survival compared to the cases, where ctDNA was deemed undetectable. The DYNAMIC study further substantiated the notion of MRD monitoring, by prospectively exploring ctDNA perioperative alterations in early-stage lung cancer patients [35]. The authors demonstrated that ctDNA quickly decays after radical tumor resection, whereas detectable MRD three days or one month after R0 resection was linked to shorter disease-free survival, but not when measured within one day. These results showed that timing of sampling matters when trying to establish a potential baseline for post-excision lung cancer monitoring. 

All the above-mentioned data clearly show that ctDNA analysis provides a multitude of benefits for real-time monitoring of MRD in postsurgical patients. ctDNA overcomes the constraints of tissue biopsies in capturing tumor heterogeneity by providing the whole clonal spectrum [36]. Another advantage is that obtaining a plasma sample is a minimally invasive process that only requires a blood withdrawal. A new shift in the era of precision oncology relies on the capacity to treat lung cancer in respect to each patient’s targetable genetic alterations, in a precise and timely manner. However, there have been several technical challenges in the detection rate and sensitivity of MRD detection since ctDNA levels in early-stage cancers and postsurgical patients are low. Today, an array of sensitive ctDNA detection systems in patients with actionable NSCLC driver gene mutations, such as the Amplification Refractory Mutation System (ARMS), the digital PCR (dPCR)/digital droplet PCR (ddPCR), or the Next-generation sequencing (NGS), have been used to overcome challenges such as insufficient DNA input amount and high costs [37]. These advantages have facilitated the conduction of several studies that are looking at using ctDNA to demonstrate the clinical utility of detecting MRD in driver-mutant NSCLC and treating recurrent disease earlier, and in the following section, we present the most impactful ones.

## 3. ctDNA in the Prognosis of EGFR-Mutant NSCLC

One of the main challenges in EGFR status assessment remains the availability as well as the quality of obtained tissue sample. It has been reported that insufficient tumor biopsy samples that yield inconclusive molecular results occur in 8–26% of patients [38]. To this significant problem, EGFR evaluation using ctDNA represents a clinically useful alternative [39]. The diagnostic accuracy of ctDNA analysis in detecting EGFR mutations in NSCLC patients’ plasma has been confirmed in several studies, where a high level of concordance compared to traditional tissue genotyping has been observed [40].

Currently, the focus has shifted towards the most commonly described mutations in *EGFR* (exon 19 deletions, p.L858R point mutation in exon 21). The frequency of these somatic activating mutations in the *EGFR* gene is estimated to be ranging from around 50% in Asian patients with NSCLC to approximately 10% in Caucasian patients [41,42]. Guidelines advocate for molecular testing for *EGFR* mutations since progression-free survival (PFS) is longer with use of *EGFR* tyrosine kinase inhibitor (TKI) monotherapy in patients with common *EGFR* mutations compared to cytotoxic systemic treatment [43,44,45]. Additionally, identification of less commonly observed alterations in *EGFR*, such as exon 19 insertions, p.L861Q, p.G719X, and p.S768I, have been associated with responsiveness to certain *EGFR* TKIs, such as osimertinib and afatinib, on a mutation-specific basis [46].

The prognostic value of ctDNA monitoring has raised the scientific interest focusing on this subpopulation since early MRD detection is crucial for extending survival [47]. Numerous studies have investigated the prognostic clinical significance of ctDNA in NSCLC patients harboring EGFR mutations. The most significant findings of the relevant studies are presented in Table 1. Interestingly, a recent pooled analysis showed that the location of metastatic site influences the diagnostic accuracy of ctDNA-based *EGFR-*mutation testing in NSCLC patients, showing higher sensitivity in patients with extrathoracic compared to intrathoracic metastases and implying a better prognosis for the latter subgroup of patients [48]. Another interesting observation is that detection of EGFR mutations in both tissue and ctDNA in NSCLC patients has been associated with higher frequency of distant metastases, as well as with significantly decreased disease-free survival (DFS). Obviously, this finding reflects the impact of MRD, which has been associated with disease relapse [49]. In another interesting study, Liu et al. showed, in a subset of EGFR-positive NSCLC patients, that allele frequency heterogeneity (AFH) defined by ctDNA is associated with poor prognosis and shorter overall survival [50]. It has also been documented that liver/bone metastases or 3–5 sites of progression of patients with EGFR mutant NSCLC, during the treatment with first line TKIs against EGFR, are associated with informative *EGFR* ctDNA testing, noting a close correlation between number and location of advanced disease with *EGFR* ctDNA. Additionally, detection of *EGFR* ctDNA mutations (with exception of T790M) is proposed as a negative prognostic factor, potentially reflecting higher burden of metastatic disease [51]. Furthermore, ctDNA copy number alterations have also been assessed as an independent predictor for shorter progression-free and overall survival [52]. It is also well documented that L858R mutation has been correlated with a shorter median OS (13.7 months) for mutation carriers versus wild-type patients (27.7 months) [53]. Other researchers used a blocker displacement amplification-derived method as a tool for MRD monitoring, to examine sequential blood samples from an *EGFR*-mutated NSCLC patient who exhibited no evidence of radiologic recurrence [37]. This patient was then treated with icotinib. Strikingly, ctDNA *EGFR* L861Q mutation was detected in a blood sample taken six months after surgery and shortly after, the patient relapsed showing multiple bone metastases in the magnetic resonance imaging scan. Cases like this clearly demonstrate the possible benefits of ctDNA-assessed MRD in clinical decision making. 

Another attempt highlighted the prognostic significance of TP53 mutations in ctDNA in advanced *EGFR*-mutant lung adenocarcinoma patients treated with gefitinib in the context of a phase 2 clinical trial [56]. Of note, NSCLC patients with TP53-mutant tumors, especially in exons 6 and 7, were significantly associated with inferior PFS and OS, compared to *EGFR*-positive patients with TP53-wild type tumors. Interestingly, the synchronous presence of both *TP53* and *EGFR* L858R mutations in ctDNA was equivalent to worse survival. All in all, current knowledge reflects the future potential of deciphering the association between blood-based *EGFR* detection and MRD dynamic monitoring, which could further support the early detection of NSCLC recurrence and the targeted individualized prognosis prediction.

## 4. ctDNA, KRAS, and NSCLC Prognosis

*KRAS* encodes a small GTPase which is part of the MAP/ERK signaling pathway. It has been characterized as proto-oncogene playing an important role in the EGF signaling cascade, by acting as downstream mediators after the binding of EGF to the EGFR [70]. According to epidemiological data, *KRAS* G12C tends to be identified in NSCLC patients with prior smoking history [71]. Smoking has been associated with the transversion of the first base (G to T), switching the wild-type glycine (GGT) to cysteine (TGT) [60]. *KRAS* mutations are found in approximately 25% of patients with adenocarcinomas, representing the most frequent genomic driver entity in NSCLC [72,73,74]. Based on the role of *KRAS* in NSCLC, novel agents have been added to the therapeutic arsenal against NSCLC and are currently used in clinical practice, such as the oral KRAS p.G12C inhibitors, small molecule drugs that were specifically designed for this mutation [29]. Identifying a *KRAS* mutation has additional value since its detection excludes the presence of *EGFR*, *ROS1*, *ALK* and *BRAF* mutations, due to the low probability of overlapping driver mutations [75,76]. Importantly, in advanced NSCLC, *KRAS* mutations have been linked to worse prognosis and shorter survival, compared to patients with wild type disease. Even in early-stage disease, *KRAS* mutations have been associated with worse DFS after complete resection of lung adenocarcinomas [77]. Therefore, *KRAS* lung tumor specific mutations are considered established prognostic biomarkers [78,79]. 

In this perspective, the use of ctDNA offers an intriguing opportunity to gain molecular information on lung cancer prognosis without the challenges of obtaining a tissue biopsy. Although driver gene mutations revealed by ctDNA profiling are similar to those of standard tissue-based genotyping, there have been some discrepancies reported, such as lower *KRAS* frequency rates in plasma [80]. Interestingly, ctDNA measurements regarding *KRAS* in perioperative patients are an emerging tool that can be used to predict the probability of disease recurrence even in *KRAS*-mutated malignancies [35,81]. Specifically in lung cancer, investigators showed in a noteworthy pre-clinical work based on a Kras^LSL-G12D^ mouse model, that ctDNA levels are measurable in mice harboring pre-malignant lung lesions detected by longitudinal micro-computed tomography (CT), suggesting ctDNA is an early-stage biomarker [82]. In addition, a study by Nygaard et al. investigated the prognostic value of plasma ctDNA in NSCLC patients with mutated *KRAS,* showing that patients with advanced NSCLC and a detectable *KRAS* ctDNA had a significantly shorter OS (4.8 months vs. 9.5 months) and PFS (3.0 months vs. 5.6 months) compared to the wild type carriers, confirming the independent negative prognostic effect of baseline *KRAS* positive mutational status [57]. Furthermore, in a prospective study, dynamic changes of *KRAS* mutant ctDNA were observed in association with treatment course in NSCLC patients, whereas the poor prognosis of patients with high levels of ctDNA was also shown [17]. This inverse relation has been confirmed by numerous studies, suggesting that ctDNA levels reflect aggressiveness as well as the potential metastatic dynamic [58]. 

On the other hand, there are some contradictory reports stating that there are no significant survival differences between patients with or without *KRAS* mutations in ctDNA, in respect to their PFS and OS [59]. However, the investigators focused solely on two *KRAS* mutations at codon 12, whereas the above-mentioned study of Nygaard et al. included six *KRAS* mutations at codon 12 and one at codon 13, making direct comparisons difficult. In addition, methylation patterns in the *KRAS* codon 12 have been examined in serum ctDNA of NSCLC patients after curative surgery [60], with no correlation being observed with survival. Interestingly, the same study revealed a high concordance between the methylation patterns of the primary tumor and serum samples, indicating that methylation assessment in peripheral blood can be a useful tool for tailoring NSCLC management. Therefore, it is well documented that comprehensive ctDNA detection in early-stage *KRAS*-mutant NSCLC and repeated postsurgical MRD monitoring could be used for the follow-up, improving the outcome of this subset of patients.

## 5. ALK Rearrangements as a Surrogate MRD Marker

*ALK* is a tyrosine kinase receptor, which is found rearranged in approximately 5% of NSCLC patients, constituting a distinct molecular and therapeutic subgroup [83]. Testing lung adenocarcinomas for *ALK* rearrangements has become standard practice since remarkable responses in *ALK* rearrangement-positive NSCLC patients treated with oral TKIs (i.e., alectinib, brigatinib, ceritinib, crizotinib, lorlatinib) have been documented [84]. Carriers of somatic *ALK* rearrangements do not respond adequately to *EGFR* TKIs despite displaying similar clinical characteristics to patients with *EGFR* mutations, including never exposure to smoke and adenocarcinoma histology [85]. According to clinical practice guidelines, *ALK* gene rearrangements, which generally harbor a conserved breakpoint in intron 19/exon 20 of *ALK*, can be detected using fluorescence in situ hybridization (FISH), immunohistochemistry (IHC), numerous NGS technologies, and targeted PCR assays [7]. 

The clinical utility of ctDNA regarding recurrence in *ALK*-rearranged NSCLC has been shown in numerous studies [86,87,88,89,90,91,92,93,94]. In patients with *ALK*-positive NSCLC, ctDNA levels have been associated with disease burden being useful as surrogate marker of MRD. In particular, it has been shown that short-term monitoring of ctDNA variations can facilitate early risk detection and improve control of *ALK*-rearranged NSCLC. Recently, in a study in which 150 NSCLC patients with *ALK* mutations were enrolled, ctDNA levels, which declined post-surgery and exhibited deviations within 7 months after surgery, were also associated with higher risk of relapse [61]. It has also been documented by analyzing ctDNA and imaging studies in patients with *ALK*-positive NSCLC who experienced disease progression while on *ALK* TKIs that there is a significant correlation between ctDNA yield and disease burden on imaging [62]. The authors noted that allelic frequency (AF) of plasma alterations is higher in cases with extrathoracic metastatic disease, especially in liver, bones, and adrenal glands. These findings come as no surprise, since ctDNA deriving from apoptotic or necrotic cancer cells primarily enters the blood stream through passive release mechanisms, suggesting that patients with oligoprogression might have negative liquid biopsies indicative of a more indolent course. The correlation of ctDNA with metastatic sites as well as clinical outcome of patients with ALK-positive disease was assessed in a recent study by Christopoulos et al. [63]. The authors showed that positive ctDNA liquid rebiopsies in *ALK*-mutated NSCLC are indicative of a more aggressive disease, which is a common observation in extracranial but rare in CNS-only progression.

In addition, ctDNA seems to be a promising marker to assess prognosis and longitudinally monitor the dynamic changes of genomic alterations in *ALK*-positive NSCLC treated with *ALK* TKIs. Characteristically, the absence of detectable ctDNA at baseline was associated with longer PFS and OS [64]. Interestingly, ctDNA clearance during *ALK*-targeted therapy was also considered a marker of better prognosis since tumors responding to the treatment release less DNA in the blood. Finally, the co-occurrence of ctDNA TP53 mutations along with *ALK* fusions was deemed as an indicator of shorter PFS [64]. Similarly, the presence of two or more *ALK* resistance mutations has been associated with worse survival outcome, probably reflecting polyclonal and resistant tumors or compound mutations [68]. Preliminary data from a phase II clinical trial (NCT03215693), in which ensartinib was evaluated, showed that higher ctDNA amount was positively correlated with tumor burden and poor PFS [65]. Finally, longitudinal monitoring of ctDNA revealed inferior PFS in ALK-positive NSCLC patients with detectable ctDNA before initiation of treatment, but also showed that an increase in ctDNA levels was prognostic of progression, preceding the radiologic determination of PD by 69 days [66].

## 6. ROS1 Rearrangements, Prognosis, and ctDNA

*ROS1* rearrangements are reported in approximately 2% of NSCLC cases, resulting in the constitutive activation of a chimeric fusion protein and the dysregulation of a tyrosine kinase-mediated signaling pathway [95]. The protein encoded from *ROS1* gene belongs to the insulin receptor family and is functionally related to ALK; however, several studies have clearly distinguished these molecules among each other. For example, *ROS1* oncogenic rearrangements are characterized by structural complexity since multiple breakpoints throughout introns 31–35 may occur, in contrast to *ALK* fusions which mainly occur at a highly conserved breakpoint found in intron 19 [96]. Furthermore, more than 15 distinct fusion partners are known to interact with *ROS1*, among which the most common include CD74 molecule (CD74), solute carrier family 34 member 2 (SLC34A2), and Golgi-associated PDZ and coiled-coil motif containing (GOPC) [97]. *ROS1* fusions are usually detected in lung tumor samples using fluorescence in-situ hybridization or NGS [98]. The spectrum of *ROS1* fusions can also be captured through ctDNA genotyping, which has been proposed as a promising approach to detect mutations that drive resistance to ROS1-directed therapies. However, the inconsistency of fusion partners and potential breakpoints make assay optimization technically challenging [86,99]. This could explain why the number of studies utilizing ctDNA as a prognostic factor in *ROS1*-rearranged NSCLC is limited. In addition, the FDA has approved crizotinib for the treatment of advanced ROS1-rearanged NSCLC patients, based on the findings of a single-arm trial, in which the objective response rate was 72% [100]. Subsequently, other tyrosine kinase inhibitors, such as ceritinib, entrectinib, and lorlatinib, have demonstrated efficacy in the treatment of metastatic ROS1-rearranged NSCLC [101,102,103].

Regarding the clinical value of ctDNA in NSCLC patients with ROS1 rearrangements, a growing number of published studies has confirmed its significance. In particular, in patients with advanced NSCLC that harbored *CD74-*, *SLC34A2-*, *SDC4-*, or *EZR-ROS1* fusions, those with isolated central nervous system progression and positive ctDNA faced higher risk of extra-CNS progression (32% vs. 7%) [104]. In addition, Dziadziuszko et al. investigated the clinical validity of an FDA approved pan-tumor liquid biopsy assay using ctDNA in identifying patients with fusion positive *ROS1* NSCLC receiving entrectinib and subsequently assessed the pre- and post-treatment genomic landscape of these patients [67]. Interestingly, the authors noted that the median duration of response was significantly shorter in ctDNA *ROS1*-fusion positive patients (5.6 vs. 17.3 months). It has also been documented that ctDNA profiling not only allows the detection of *ALK/ROS1* fusions, but also enables the identification of resistance mutations, such as *ROS1* G2032R, in patients treated with TKIs, whereas the absence of ctDNA mutations has been associated with improved OS [68]. Therefore, current approaches enable us to detect and quantify *ROS1* rearrangements and other somatic mutations in plasma ctDNA, including driver mutation-mediated drug resistance, paving the way for its application in monitoring tumor dynamics in a clinical setting.

## 7. ctDNA in *BRAF-*Mutant NSCLC

*BRAF* mutations, which are detected in 1–2% of patients with NSCLC, with the most common resulting in the substitution of glutamate with valine at codon 600 (V600E), have become a promising therapeutic target [75]. The inhibition of *BRAF* V600E and its downstream effector *MEK* with oral inhibitors (dabrafenib and trametinib, respectively) is the most effective strategy in terms of activity and efficacy in metastatic *BRAF* V600E NSCLC [105]. If the combination is not tolerated, single-agent vemurafenib or dabrafenib are also available treatment options [75,106]. However, acquired resistance is a phenomenon commonly observed after the administration of targeted therapies; therefore, liquid biopsy approaches could be utilized for the assessment of these resistance mutations that emerge, ensuring the avoidance of repeated biopsies in some cases.

In a prospective study, in which *BRAF* V600E mutant NSCLC patients were treated with *BRAF/MEK* inhibitors, amplicon-based NGS analysis on ctDNA obtained at progression was performed [107]. Interestingly, the longitudinal evaluation of the molecular alterations in *BRAF* was in line with the course of the disease, with ctDNA levels rising at disease progression. Additionally, the detection of ctDNA mutations was highly dependent on disease dissemination, with ctDNA being detectable in cases of systemic metastasis and not in cases of intrathoracic or brain disease. For the latter cases, a case report noted that NGS analysis of cerebrospinal fluid ctDNA in *BRAF*-mutant NSCLC patients with brain metastasis, may potentially provide more accurate information about intracranial lesions than blood serum ctDNA, due to the blood-brain barrier [108]. Furthermore, detection of *BRAF* mutations that activate *MAPK* and *PI3K* signaling pathway effectors in ctDNA has also been correlated with patient outcomes. Particularly, ctDNA *BRAF* alterations detected during radiological disease progression were associated with poor overall survival [109]. In this context, monitoring of BRAF-mutant alleles could act as an identifier of residual disease and an early indicator of progression, as it has been displayed in *BRAF*-mutant melanoma [110].

## 8. Prognostic Significance of ctDNA in RET-Rearranged NSCLC

*RET* is also a known proto-oncogene that affects cell proliferation and differentiation. In NSCLCs, rearrangements may occur between the *RET* gene and other genomic regions, especially Kinesin Family Member 5B (*KIF5B*), Nuclear Receptor Coactivator 4 (*NCOA4*), and Coiled-Coil Domain Containing 6 (*CCDC6*), which are the most common and the best characterized upstream fusion partners [111]. Approximately 1–2% of NSCLC patients harbor a fusion in the *RET* proto-oncogene. They are more frequent in patients with adenocarcinoma histology who have none or minimal history of tobacco use. Unlike *ALK* and *ROS1* rearrangements that were previously discussed, *RET* fusion genes cannot be adequately detected by IHC; instead NGS, FISH, and RT-PCR can be used as alternative diagnostic tools [112].

Current guidelines recommend testing for RET rearrangements in eligible patients with metastatic NSCLC, based on a clinical trial that led to the FDA approvals of selective *RET* inhibitors selpercatinib and pralsetinib [113,114]. Towards this direction, plasma-based ctDNA analysis may be particularly useful. Interestingly, using a comprehensive NGS assay in 14,639 patients with metastatic NSCLC, ctDNA analysis revealed 125 *RET* alterations, with *KIF5B-RET* fusions being highly specific for NSCLC [115]. Furthermore, a recent case report described a patient with *KIF5B-RET* fusion-positive advanced NSCLC, in whom ctDNA assessment identified a previously undetectable RET-KIF5B fusion during treatment with an oral *RET*-inhibitor [116]. The re-emergence of the activating fusion prompted early CT imaging and resulted in immediate detection of disease progression, highlighting the benefit of serial liquid biopsies as a useful, minimally invasive method to detect relapse.

## 9. MRD, MET Amplifications, and MET Exon 14 Skipping Variants in NSCLC

Tumorigenesis usually involves the activation of the growth-promoting gene *MET*, while the relevant protein is another targetable molecule with available targeted therapies [117]. Interestingly, a somatic genomic alteration that results in loss of *MET* exon 14 occurs in NSCLC, leading to promotion of tumor cell growth, survival, and invasion [118,119]. NGS-based testing is the primary method for detection of these events in contrast to IHC, which is not a suitable method for the detection of *MET*ex14 skipping variants. For NGS-based results, a copy number greater than ten is consistent with high-level *MET* amplification, which is a driver event in lung cancer [120]. Notably, available targeted agents with activity against high-level *MET* amplifications are currently available, namely crizotinib [121], capmatinib [120], and tepotinib [122].

Attention has been called to the detection of *MET* mutations in plasma ctDNA [123]. For example, in a comprehensive genomic profiling study of ctDNA from 1552 NSCLC patients, *MET*ex14 skipping mutations were detected in 1.9% of cases. Among them, three cases stood apart that harbored additional activating single-nucleotide variants of *MET* (p. L1195V, p. D1228H, p. Y1230C) [124]. Regarding the clinical associations of detecting *MET* ctDNA aberrations, they have been correlated with poor prognostic indicators, such as bone metastases, co-existing somatic genomic alterations, and a worse OS and shorter median time to recurrence or metastases in patients with diverse malignancies, among them NSCLC [69]. It seems that the potentials of liquid biopsy and especially the assessment of ctDNA for optimizing the risk assessment of disease recurrence and treatment response has become apparent.

## 10. Conclusions

Precision medicine requires the accurate molecular profiling of patients with NSCLC in order to secure the most appropriate therapeutic interventions. In mutation-driven NSCLC, the use of liquid biopsies is highlighted as an emerging trend in the era of personalized medicine. In this perspective, the potential of ctDNA is better displayed due to the recent FDA approval of *EGFR* mutational assessment on plasma ctDNA in patients with advanced NSCLC. Furthermore, the results of the phase II DYNAMIC trial that were recently announced in ASCO 2022, which was the first study to use ctDNA to direct adjuvant therapy in colon cancer, highlighted the ability to implement a ctDNA-guided management [125]. Indeed, serial ctDNA analysis in biofluids is a minimally invasive approach for the detection and tracking of cancer driver mutations, for monitoring therapeutic response to personalized targeted therapies, and for identifying minimal residual disease, allowing for a more precise assessment of disease recurrence risk and patient selection for adjuvant therapy. These are all clinically relevant parameters that impact oncologic lung cancer management in a real-world setting. Overall, the reviewed studies provide evidence that monitoring the status of druggable genetic driver mutations in ctDNA can be used as a promising prognostic biomarker associated with targeted NSCLC therapeutic options. We also collectively highlight the importance of early identification of residual disease that allows us to target and stratify patients according to their recurrence risk in a more individualized manner.

## Figures and Tables

**Table 1:** Studies and findings focusing on prognostic ctDNA-based analyses in patients with oncogene-driven NSCLC.

Study (Year)	Inclusion Criteria	*n*	Sample	Detection Method	Follow-Up	Prognostic Relevance
Guo et al., 2021 [49]	Stage I–III EGFR-mutated NSCLC	174	Blood	Real-time PCR ARMS	5 years	5-year survival rate ctDNA EGFR mut+ 18.5% vs. EGFR mut- 76.9% median OS ctDNA EGFR mut+ 29.00 ± 2.55 m vs. EGFR mut- not reached ctDNA EGFR mut+ independent prognostic risk factor for DFS, OS ctDNA EGFR mut+ patients shorter DFS of 19.00 ± 2.50 m Probability of developing distant metastasis ctDNA EGFR mut+ 81.5% vs. EGFR mut- 25.2%
Liu et al., 2019 [50]	Advanced EGFR-mutated NSCLC under first-line TKIs	259	Blood	Targeted NGS	Jan 2012 to December 2018	EGFR-TKIs cohort: presence of allele frequency heterogeneity in ctDNA significantly associated with shorter OS
Pender et al., 2020 [51]	Advanced EGFR-mutated NSCLC	177	Blood	ddPCR	February 2018 to March 2019	Median OS ctDNA EGFR mut+ patients 8.18 m vs. EGFR mut- 25.3 m EGFR mut and ≥6 sites of progression = higher risk of death
Yu et al., 2020 [54]	Metastatic EGFR-mutated NSCLC treated with osimertinib/bevacizumab	49	Blood	ddPCR	August 2016 to May 2018	Persistent detection of EGFR mut ctDNA at six weeks associated with shorter median PFS (16.2 m vs. 9.8 m) and median OS (10.1 m)
Buder et al., 2021 [55]	Advanced EGFR-mutated lung adenocarcinoma, PD under TKI	43	Blood	ddPCR	August 2015 and January 2019	Somatic copy-number alterations in ctDNA independent predictor for shorter PFS and OS
Yu et al., 2021 [56]	Advanced treatment-naïve EGFR-mutant lung adenocarcinoma treated with gefitinib	180	Blood	ddPCR	December 2014 to June 2019	PFS and OS of patients with ctDNA TP53-wt tumors significantly longer vs. TP53-mut tumors (OS: 21.2 m vs. 32.0 m; PFS: 8.4 m vs. 12.81 m) Patients with ctDNA TP53 and EGFR exon 19 mut significantly longer PFS and OS vs. TP53 and EGFR L858R mutations (26.8 m vs. 21.5 m)
Karachaliou et al., 2015 [53]	Advanced EGFR mutated NSCLC treated with erlotinib or chemo	97	Blood	T-PCR (TaqMan) assay	2007 to 2011	Median OS in ctDNA L858R mut+ 13.7 m vs. exon 19 del 30.0 m ctDNA L858R mut marker of shorter OS and PFS
Xu et al., 2022 [37]	NSCLC Stage IB (T2N0M0) EGFR L861Q-mutated	1	Blood	Personalized Analysis of Cancer (blocker displacement amplification)	March 2020 to March 2021	ctDNA *EGFR* L861Q mutation detected in blood sample two months prior to radiologically identified metastasis
Nygaard et al., 2013 [57]	NSCLC stage III orIV, no previous chemo, PS ≤ 2 and age > 18 years	246	Blood	ARMS-qPCR	2007–2010	Median OS ctDNA *KRAS* mut+ 4.8 m vs. KRAS wt 9.5 m Median PFS ctDNA *KRAS* mut+ 3.0 m vs. KRAS wt 5.6 m Independent prognostic value of *KRAS* in OS
Gautschi et al., 2007 [58]	NSCLC	180	Blood	RFLP–PCR	April 2001 to December 2004	OS ctDNA *KRAS* mut+ significantly worse vs. *KRAS* wt
Camps et al., 2011 [59]	NSCLC stage IIIB or IV prior to cisplatin/docetaxel chemo	308	Blood	Fluorogenic RT-PCR	9.68 months	PFS similar between KRAS wt and KRAS mut+ (5.77 m vs. 5.43 m) OS similar for both KRAS genotype groups (9.07 m vs. 10.03 m)
Ramirez et al., 2013 [60]	NSCLC aftercurative surgery	50	Blood	PCR	October 1998 to September 1999	Significantly worse survival for serum KRAS mut+ patients
Li et al., 2020 [61]	ALK-positive NSCLC	150	Blood	Not Available	Not Available	ctDNA baseline 57 ng/mL vs. 30 ng/mL post-surgery ctDNA deviations within 7 months of surgery significant predictors for RFS
Zhang et al., 2020 [62]	ALK-positive NSCLC, PD under TKI	75	Blood	NGS	March 2016 to March 2019	Significant correlation between ctDNA burden and disease burden as assessed by RECIST, volumetric segmentation analysis, quantitative tallying of organ-specific metastasis
Christopoulos et al., 2021 [63]	Consecutive TKI-treated ALK-positive NSCLC	56	Blood	NGS	2014 to 2019	OS of ctDNA ALK mut+ patients with extracranial progression shorter (mean 52 vs. 69 m) ctDNA detectability not associated with outcome of patients with CNS-only progression
Kwon et al., 2020 [64]	ALK-positive advanced NSCLC	92	Blood	NGS	April 2015 to July 2019	Un-detectable ctDNA at baseline associated with longer median PFS (36.1 vs. 11.6 m) and OS (not reached vs. 27.9 m) ctDNA clearance at two months longer median PFS (25.4 vs. 13.9 m) and OS (not reached vs. 25.7 m) Co-occurring ctDNA *TP53* mut at baseline shorter PFS (7.0 vs. 12.5 m)
Yang et al., 2020 [65]	Stage IIIB/IV ALK-positive NSCLC, PD after crizotinib	182	Blood	NGS	September 2017 to July 2019	Higher ctDNA amount associated with liver/bone metastases, TP53 mut, and tumor burden High ctDNA levels and TP53 mut at baseline associated with poor PFS
Madsen et al., 2020 [66]	ALK-positive non-squamous NSCLC	24	Blood	ddPCR	December 2015 to November 2018	Detectable ctDNA prior to treatment worse median PFS (8.7 vs. 15.2 m) ctDNA within two months after treatment predicted inferior median PFS (4.6 vs. 14.5 m)
Dziadziuszko et al., 2022 [67]	Advanced *NTRK* or *ROS1*-fusion protein NSCLC	85	Blood	NGS	November 2015 to May 2018	Median duration of response to erlotinib significantly differed between ctDNA *ROS1* mut+ 5.6 vs. *ROS1* mut- vs. 17.3 m
Mezquita et al., 2020 [68]	ALK- and ROS1- fusion–positive advanced NSCLC	128 (101 ALK+, 27 ROS+)	Blood	NGS	October 2015 to August 2018	Absence of *ALK* ctDNA mut at TKI failure associated with prolonged median OS (44.1 vs. 105.7 m) *ROS1* G2032R predictive of rapid PD (<3 months) under TKI
Ikeda et al., 2018 [69]	Cancer Patients	102 (12 MET+)	Blood	NGS	June 2014 to July 2016	ctDNA MET alterations correlated with bone metastasis and TP53/PTEN abnormalities *MET* mut+ shorter median time to metastasis/recurrence (1.0 m vs. 10.4 m) and poorer survival (30.6 m vs. 58.4 m)

PCR: polymerase chain reaction, ddPCR: droplet digital polymerase chain reaction ARMS: Amplification Refractory Mutation System, PFS: progression free survival, RFS: recurrence free survival, OS: overall survival, PD: progressive disease, PS: performance status, Chemo: chemotherapy.

## Data Availability

Not applicable.

## References

[1] Yates L.R., Seoane J., Le Tourneau C., Siu L.L., Marais R., Michiels S., Soria J.C., Campbell P., Normanno N., Scarpa A. (2018). The European Society for Medical Oncology (ESMO) Precision Medicine Glossary. Ann. Oncol..

[2] Pfohl U., Pflaume A., Regenbrecht M., Finkler S., Adelmann Q.G., Reinhard C., Regenbrecht C.R.A., Wedeken L. (2021). Precision Oncology Beyond Genomics: The Future Is Here—It Is Just Not Evenly Distributed. Cells.

[3] Sung H., Ferlay J., Siegel R.L., Laversanne M., Soerjomataram I., Jemal A., Bray F. (2021). Global cancer statistics 2020: GLOBOCAN estimates of incidence and mortality worldwide for 36 cancers in 185 countries. CA Cancer J. Clin..

[4] Inamura K. (2017). Lung Cancer: Understanding Its Molecular Pathology and the 2015 WHO Classification. Front. Oncol..

[5] Nicholson A.G., Tsao M.S., Beasley M.B., Borczuk A.C., Brambilla E., Cooper W.A., Dacic S., Jain D., Kerr K.M., Lantuejoul S. (2022). The 2021 WHO Classification of Lung Tumors: Impact of Advances Since 2015. J. Thorac. Oncol..

[6] Chaft J.E., Rimner A., Weder W., Azzoli C.G., Kris M.G., Cascone T. (2021). Evolution of systemic therapy for stages I–III non-metastatic non-small-cell lung cancer. Nat. Rev. Clin. Oncol..

[7] National Comprehensive Cancer Network NCCN Clinical Practice Guidelines in Oncology Non-Small Cell Lung Cancer. https://www.nccn.org/professionals/physician_gls/pdf/nscl.pdf.

[8] Uramoto H., Tanaka F. (2014). Recurrence after surgery in patients with NSCLC. Transl. Lung Cancer Res..

[9] Woodard G.A., Wang S.X., Kratz J.R., Zoon-Besselink C.T., Chiang C.Y., Gubens M.A., Jahan T.M., Blakely C.M., Jones K.D., Mann M.J. (2018). Adjuvant Chemotherapy Guided by Molecular Profiling and Improved Outcomes in Early Stage, Non-Small-Cell Lung Cancer. Clin. Lung Cancer.

[10] Aguirre-Ghiso J.A., Sosa M.S. (2018). Emerging Topics on Disseminated Cancer Cell Dormancy and the Paradigm of Metastasis. Annu. Rev. Cancer Biol..

[11] Fluegen G., Avivar-Valderas A., Wang Y., Padgen M.R., Williams J.K., Nobre A.R., Calvo V., Cheung J.F., Bravo-Cordero J.J., Entenberg D. (2017). Phenotypic heterogeneity of disseminated tumour cells is preset by primary tumour hypoxic microenvironments. Nat. Cell Biol..

[12] Sosa M.S., Bragado P., Aguirre-Ghiso J.A. (2014). Mechanisms of disseminated cancer cell dormancy: An awakening field. Nat. Rev. Cancer.

[13] Giancotti F.G. (2013). Mechanisms governing metastatic dormancy and reactivation. Cell.

[14] Pantel K., Brakenhoff R.H. (2004). Dissecting the metastatic cascade. Nat. Rev. Cancer.

[15] Fares J., Kanojia D., Rashidi A., Ahmed A.U., Balyasnikova I.V., Lesniak M.S. (2019). Diagnostic Clinical Trials in Breast Cancer Brain Metastases: Barriers and Innovations. Clin. Breast Cancer.

[16] Shaw J.A., Guttery D.S., Hills A., Fernandez-Garcia D., Page K., Rosales B.M., Goddard K.S., Hastings R.K., Luo J., Ogle O. (2017). Mutation Analysis of Cell-Free DNA and Single Circulating Tumor Cells in Metastatic Breast Cancer Patients with High Circulating Tumor Cell Counts. Clin. Cancer Res..

[17] Abbosh C., Birkbak N.J., Wilson G.A., Jamal-Hanjani M., Constantin T., Salari R., Le Quesne J., Moore D.A., Veeriah S., Rosenthal R. (2017). Phylogenetic ctDNA analysis depicts early-stage lung cancer evolution. Nature.

[18] Zill O.A., Banks K.C., Fairclough S.R., Mortimer S.A., Vowles J.V., Mokhtari R., Gandara D.R., Mack P.C., Odegaard J.I., Nagy R.J. (2018). The Landscape of Actionable Genomic Alterations in Cell-Free Circulating Tumor DNA from 21,807 Advanced Cancer Patients. Clin. Cancer Res..

[19] Joosse S.A., Gorges T.M., Pantel K. (2015). Biology, detection, and clinical implications of circulating tumor cells. EMBO Mol. Med..

[20] US Food and Drug Administration. 2016. Cobas EGFR Mutation Test v2|FDA. https://www.fda.gov/drugs/resources-information-approved-drugs/cobas-egfr-mutation-test-v2.

[21] European Medicines Agency. 2014. Iressa: EPAR—Product Information. https://www.ema.europa.eu/en/medicines/human/EPAR/iressa.

[22] Wu C.Y., Lee C.L., Wu C.F., Fu J.Y., Yang C.T., Wen C.T., Liu Y.H., Liu H.P., Hsieh J.C.H. (2020). Circulating Tumor Cells as a Tool of Minimal Residual Disease Can Predict Lung Cancer Recurrence: A longitudinal, Prospective Trial. Diagnostics.

[23] Lin E.C. (2010). Radiation Risk From Medical Imaging. Mayo Clin. Proc..

[24] Bardelli A., Pantel K. (2017). Liquid Biopsies, What We Do Not Know (Yet). Cancer Cell.

[25] Arbour K.C., Riely G.J. (2019). Systemic Therapy for Locally Advanced and Metastatic Non-Small Cell Lung Cancer: A Review. JAMA.

[26] Pao W., Girard N. (2011). New driver mutations in non-small-cell lung cancer. Lancet. Oncol..

[27] Riudavets M., Sullivan I., Abdayem P., Planchard D. (2021). Targeting HER2 in non-small-cell lung cancer (NSCLC): A glimpse of hope? An updated review on therapeutic strategies in NSCLC harbouring HER2 alterations. ESMO Open.

[28] Collisson E.A., Campbell J.D., Brooks A.N., Berger A.H., Lee W., Chmielecki J., Beer D.G., Cope L., Creighton C.J., Danilova L. (2014). Comprehensive molecular profiling of lung adenocarcinoma. Nature.

[29] Skoulidis F., Li B.T., Dy G.K., Price T.J., Falchook G.S., Wolf J., Italiano A., Schuler M., Borghaei H., Barlesi F. (2021). Sotorasib for Lung Cancers with KRAS p.G12C Mutation. N. Engl. J. Med..

[30] Jänne P.A., Riely G.J., Gadgeel S.M., Heist R.S., Ou S.-H.I., Pacheco J.M., Johnson M.L., Sabari J.K., Leventakos K., Yau E. (2022). Adagrasib in Non–Small-Cell Lung Cancer Harboring a KRAS G12C Mutation. N. Engl. J. Med..

[31] Keppens C., Palma J.F., Das P.M., Scudder S., Wen W., Normanno N., van Krieken J.H., Sacco A., Fenizia F., Gonzalez de Castro D. (2018). Detection of EGFR Variants in Plasma: A Multilaboratory Comparison of a Real-Time PCR EGFR Mutation Test in Europe. J. Mol. Diagnostics.

[32] Shagin D.A., Shagina I.A., Zaretsky A.R., Barsova E.V., Kelmanson I.V., Lukyanov S., Chudakov D.M., Shugay M. (2017). A high-throughput assay for quantitative measurement of PCR errors. Sci. Rep..

[33] Pignon J.P., Tribodet H., Scagliotti G.V., Douillard J.Y., Shepherd F.A., Stephens R.J., Dunant A., Torri V., Rosell R., Seymour L. (2008). undefined Lung adjuvant cisplatin evaluation: A pooled analysis by the LACE Collaborative Group. Da-tabase of Abstracts of Reviews of Effects (DARE): Quality-Assessed Reviews.

[34] Chaudhuri A.A., Chabon J.J., Lovejoy A.F., Newman A.M., Stehr H., Azad T.D., Khodadoust M.S., Esfahani M.S., Liu C.L., Zhou L. (2017). Early detection of molecular residual disease in localized lung cancer by circulating tumor DNA profiling. Cancer Discov..

[35] Chen K., Zhao H., Shi Y., Yang F., Wang L.T., Kang G., Nie Y., Wang J. (2019). Perioperative dynamic changes in circulating tumor DNA in patients with lung cancer (Dynamic). Clin. Cancer Res..

[36] Gremel G., Lee R.J., Girotti M.R., Mandal A.K., Valpione S., Garner G., Ayub M., Wood S., Rothwell D.G., Fusi A. (2016). Distinct subclonal tumour responses to therapy revealed by circulating cell-free DNA. Ann. Oncol. Off. J. Eur. Soc. Med. Oncol..

[37] Xu J., Pu Y., Lin R., Xiao S., Fu Y., Wang T. (2022). PEAC: An Ultrasensitive and Cost-Effective MRD Detection System in Non-small Cell Lung Cancer Using Plasma Specimen. Front. Med..

[38] Smeltzer M.P., Wynes M.W., Lantuejoul S., Soo R., Ramalingam S.S., Varella-Garcia M., Meadows Taylor M., Richeimer K., Wood K., Howell K.E. (2020). The International Association for the Study of Lung Cancer Global Survey on Molecular Testing in Lung Cancer. J. Thorac. Oncol..

[39] Kunimasa K., Nishino K., Sato Y., Mori M., Ihara S., Suzuki H., Nagatomo I., Kumagai T., Morishima T., Imamura F. (2022). Fragment size and dynamics of EGFR-mutated tumor-derived DNA provide prognostic information regarding EGFR-TKI efficacy in patients with EGFR-mutated NSCLC. Sci. Rep..

[40] Thress K.S., Brant R., Carr T.H., Dearden S., Jenkins S., Brown H., Hammett T., Cantarini M., Barrett J.C. (2015). EGFR mutation detection in ctDNA from NSCLC patient plasma: A cross-platform comparison of leading technologies to support the clinical development of AZD9291. Lung Cancer.

[41] Melosky B., Kambartel K., Häntschel M., Bennetts M., Nickens D.J., Brinkmann J., Kayser A., Moran M., Cappuzzo F. (2022). Worldwide Prevalence of Epidermal Growth Factor Receptor Mutations in Non-Small Cell Lung Cancer: A Meta-Analysis. Mol. Diagnosis Ther..

[42] Zhang Y.L., Yuan J.Q., Wang K.F., Fu X.H., Han X.R., Threapleton D., Yang Z.Y., Mao C., Tang J.L. (2016). The prevalence of EGFR mutation in patients with non-small cell lung cancer: A systematic review and meta-analysis. Oncotarget.

[43] Rosell R., Carcereny E., Gervais R., Vergnenegre A., Massuti B., Felip E., Palmero R., Garcia-Gomez R., Pallares C., Sanchez J.M. (2012). Erlotinib versus standard chemotherapy as first-line treatment for European patients with advanced EGFR mutation-positive non-small-cell lung cancer (EURTAC): A multicentre, open-label, randomised phase 3 trial. Lancet. Oncol..

[44] Yang J.C.H., Wu Y.L., Schuler M., Sebastian M., Popat S., Yamamoto N., Zhou C., Hu C.P., O’Byrne K., Feng J. (2015). Afatinib versus cisplatin-based chemotherapy for EGFR mutation-positive lung adenocarcinoma (LUX-Lung 3 and LUX-Lung 6): Analysis of overall survival data from two randomised, phase 3 trials. Lancet. Oncol..

[45] Sequist L.V., Yang J.C.H., Yamamoto N., O’Byrne K., Hirsh V., Mok T., Geater S.L., Orlov S., Tsai C.M., Boyer M. (2013). Phase III study of afatinib or cisplatin plus pemetrexed in patients with metastatic lung adenocarcinoma with EGFR mutations. J. Clin. Oncol..

[46] Chiu C.H., Yang C.T., Shih J.Y., Huang M.S., Su W.C., Lai R.S., Wang C.C., Hsiao S.H., Lin Y.C., Ho C.L. (2015). Epidermal Growth Factor Receptor Tyrosine Kinase Inhibitor Treatment Response in Advanced Lung Adenocarcinomas with G719X/L861Q/S768I Mutations. J. Thorac. Oncol..

[47] Chabon J.J., Hamilton E.G., Kurtz D.M., Esfahani M.S., Moding E.J., Stehr H., Schroers-Martin J., Nabet B.Y., Chen B., Chaudhuri A.A. (2020). Integrating genomic features for noninvasive early lung cancer detection. Nature.

[48] Passiglia F., Rizzo S., Rolfo C., Galvano A., Bronte E., Incorvaia L., Listi A., Barraco N., Castiglia M., Calo V. (2018). Metastatic Site Location Influences the Diagnostic Accuracy of ctDNA EGFR- Mutation Testing in NSCLC Patients: A Pooled Analysis. Curr. Cancer Drug Targets.

[49] Guo K., Shao C., Han L., Liu H., Ma Z., Yang Y., Feng Y., Pan M., Santarpia M., Carmo-Fonseca M. (2021). Detection of epidermal growth factor receptor (EGFR) mutations from preoperative circulating tumor DNA (ctDNA) as a prognostic predictor for stage I–III non-small cell lung cancer (NSCLC) patients with baseline tissue EGFR mutations. Transl. Lung Cancer Res..

[50] Liu Z., Xie Z., Zhao S., Ye D., Cai X., Cheng B., Li C., Xiong S., Li J., Liang H. (2019). Presence of allele frequency heterogeneity defined by ctDNA profiling predicts unfavorable overall survival of NSCLC. Transl. Lung Cancer Res..

[51] Pender A., Hughesman C., Law E., Kristanti A., McNeil K., Wong S., Tucker T., Bosdet I., Young S., Laskin J. (2020). EGFR circulating tumour DNA testing: Identification of predictors of ctDNA detection and implications for survival outcomes. Transl. Lung Cancer Res..

[52] Mok T., Wu Y.L., Lee J.S., Yu C.J., Sriuranpong V., Sandoval-Tan J., Ladrera G., Thongprasert S., Srimuninnimit V., Liao M. (2015). Detection and dynamic changes of EGFR mutations from circulating tumor DNA as a predictor of survival outcomes in NSCLC Patients treated with first-line intercalated erlotinib and chemotherapy. Clin. Cancer Res..

[53] Karachaliou N., Mayo-De Las Casas C., Queralt C., De Aguirre I., Melloni B., Cardenal F., Garcia-Gomez R., Massuti B., Sánchez J.M., Porta R. (2015). Association of EGFR L858R Mutation in Circulating Free DNA With Survival in the EURTAC Trial. JAMA Oncol..

[54] Yu H.A., Schoenfeld A.J., Makhnin A., Kim R., Rizvi H., Tsui D., Falcon C., Houck-Loomis B., Meng F., Yang J.L. (2020). Effect of Osimertinib and Bevacizumab on Progression-Free Survival for Patients With Metastatic EGFR-Mutant Lung Cancers: A Phase 1/2 Single-Group Open-Label Trial. JAMA Oncol..

[55] Buder A., Heitzer E., Waldispühl-Geigl J., Weber S., Moser T., Hochmair M.J., Hackner K., Errhalt P., Setinek U., Filipits M. (2021). Somatic copy-number alterations in plasma circulating tumor dna from advanced egfr-mutated lung adenocarcinoma patients. Biomolecules.

[56] Yu R., Bai H., Li T., Gao B., Han J., Chang G., Zhang P., Fei K., He X., Wang J. (2021). TP53 mutations in circulating tumor DNA in advanced epidermal growth factor receptor-mutant lung adenocarcinoma patients treated with gefitinib. Transl. Oncol..

[57] Nygaard A.D., Garm Spindler K.L., Pallisgaard N., Andersen R.F., Jakobsen A. (2013). The prognostic value of KRAS mutated plasma DNA in advanced non-small cell lung cancer. Lung Cancer.

[58] Gautschi O., Huegli B., Ziegler A., Gugger M., Heighway J., Ratschiller D., Mack P.C., Gumerlock P.H., Kung H.J., Stahel R.A. (2007). Origin and prognostic value of circulating KRAS mutations in lung cancer patients. Cancer Lett..

[59] Camps C., Jantus-Lewintre E., Cabrera A., Blasco A., Sanmartín E., Gallach S., Caballero C., Del Pozo N., Rosell R., Guijarro R. (2011). The identification of KRAS mutations at codon 12 in plasma DNA is not a prognostic factor in advanced non-small cell lung cancer patients. Lung Cancer.

[60] Ramirez J.L., Sarries C., De Castro P.L., Roig B., Queralt C., Escuin D., De Aguirre I., Sanchez J.M., Manzano J.L., Margelí M. (2003). Methylation patterns and K-ras mutations in tumor and paired serum of resected non-small-cell lung cancer patients. Cancer Lett..

[61] Li J., Dong W., Liu L.N., Huang Y.J., Xiao M.F. (2020). Liquid biopsy for ALK-positive early non-small-cell lung cancer predicts disease relapse. Future Oncol..

[62] Zhang E.W., Dagogo-Jack I., Kuo A., Rooney M.M., Shaw A.T., Digumarthy S.R. (2020). Association between circulating tumor DNA burden and disease burden in patients with ALK-positive lung cancer. Cancer.

[63] Christopoulos P., Dietz S., Angeles A.K., Rheinheimer S., Kazdal D., Volckmar A.L., Janke F., Endris V., Meister M., Kriegsmann M. (2021). Earlier extracranial progression and shorter survival in ALK- rearranged lung cancer with positive liquid rebiopsies. Transl. Lung Cancer Res..

[64] Kwon M., Ku B.M., Park S., Jung H.A., Sun J.-M., Lee S.-H., Ahn J.S., Park K., Ahn M.-J. (2020). Longitudinal monitoring by next generation sequencing of plasma cell-free DNA in ALK-rearranged non-small cell lung cancer (NSCLC) patients treated with ALK tyrosine kinase inhibitors. Cancer Med..

[65] Yang Y., Huang J., Wang T., Zhou J., Zheng J., Feng J., Zhuang W., Chen J., Zhao J., Zhong W. (2020). Abstract 2997: Longitudinal circulating tumor DNA (ctDNA) analysis predicts response and reveals the resistance mechanisms of ensartinib in ALK+ NSCLC patients (pts) progressed on crizotinib: Updated analysis of a phase II clinical trial. Cancer Res..

[66] Madsen A.T., Winther-Larsen A., McCulloch T., Meldgaard P., Sorensen B.S. (2020). Genomic Profiling of Circulating Tumor DNA Predicts Outcome and Demonstrates Tumor Evolution in ALK-Positive Non-Small Cell Lung Cancer Patients. Cancers.

[67] Dziadziuszko R., Hung T., Wang K., Choeurng V., Drilon A., Doebele R.C., Barlesi F., Wu C., Dennis L., Skoletsky J. (2022). Pre- and post-treatment blood-based genomic landscape of patients with ROS1 or NTRK fusion-positive solid tumours treated with entrectinib. Mol. Oncol..

[68] Mezquita L., Swalduz A., Jovelet C., Ortiz-Cuaran S., Howarth K., Planchard D., Avrillon V., Recondo G., Marteau S., Benitez J.C. (2020). Clinical Relevance of an Amplicon-Based Liquid Biopsy for Detecting ALK and ROS1 Fusion and Resistance Mutations in Patients with Non–Small-Cell Lung Cancer. JCO Precis. Oncol..

[69] Ikeda S., Schwaederle M., Mohindra M., Fontes Jardim D.L., Kurzrock R. (2018). MET alterations detected in blood-derived circulating tumor DNA correlate with bone metastases and poor prognosis. J. Hematol. Oncol..

[70] Wee P., Wang Z. (2017). Epidermal Growth Factor Receptor Cell Proliferation Signaling Pathways. Cancers.

[71] Wang X., Ricciuti B., Nguyen T., Li X., Rabin M.S., Awad M.M., Lin X., Johnson B.E., Christiani D.C. (2021). Association between Smoking History and Tumor Mutation Burden in Advanced Non-Small Cell Lung Cancer. Cancer Res..

[72] Eberhard D.A., Johnson B.E., Amler L.C., Goddard A.D., Heldens S.L., Herbst R.S., Ince W.L., Jänne P.A., Januario T., Johnson D.H. (2005). Mutations in the epidermal growth factor receptor and in KRAS are predictive and prognostic indicators in patients with non-small-cell lung cancer treated with chemotherapy alone and in combination with erlotinib. J. Clin. Oncol..

[73] Biernacka A., Tsongalis P.D., Peterson J.D., de Abreu F.B., Black C.C., Gutmann E.J., Liu X., Tafe L.J., Amos C.I., Tsongalis G.J. (2016). The potential utility of re-mining results of somatic mutation testing: KRAS status in lung adenocarcinoma. Cancer Genet..

[74] Rakhit C.P., Ottolini B., Jones C., Pringle J.H., Shaw J.A., Martins L.M. (2017). Peptide nucleic acid clamping to improve the sensitivity of Ion Torrent-based detection of an oncogenic mutation in KRAS. Matters.

[75] Planchard D., Besse B., Groen H.J.M., Souquet P.J., Quoix E., Baik C.S., Barlesi F., Kim T.M., Mazieres J., Novello S. (2016). Dabrafenib plus trametinib in patients with previously treated BRAF(V600E)-mutant metastatic non-small cell lung cancer: An open-label, multicentre phase 2 trial. Lancet. Oncol..

[76] Sholl L.M., Aisner D.L., Varella-Garcia M., Berry L.D., Dias-Santagata D., Wistuba I.I., Chen H., Fujimoto J., Kugler K., Franklin W.A. (2015). Multi-institutional Oncogenic Driver Mutation Analysis in Lung Adenocarcinoma: The Lung Cancer Mutation Consortium Experience. J. Thorac. Oncol..

[77] Jones G.D., Caso R., Tan K.S., Mastrogiacomo B., Sanchez-Vega F., Liu Y., Connolly J.G., Murciano-Goroff Y.R., Bott M.J., Adusumilli P.S. (2021). KRASG12Cmutation is associated with increased risk of recurrence in surgically resected lung adenocarcinoma. Clin. Cancer Res..

[78] Slebos R.J.C., Kibbelaar R.E., Dalesio O., Kooistra A., Stam J., Meijer C.J.L.M., Wagenaar S.S., Vanderschueren R.G.J.R.A., van Zandwijk N., Mooi W.J. (1990). K-ras oncogene activation as a prognostic marker in adenocarcinoma of the lung. N. Engl. J. Med..

[79] Tsao M.S., Aviel-Ronen S., Ding K., Lau D., Liu N., Sakurada A., Whitehead M., Zhu C.Q., Livingston R., Johnson D.H. (2007). Prognostic and predictive importance of p53 and RAS for adjuvant chemotherapy in non small-cell lung cancer. J. Clin. Oncol..

[80] Zhang Y., Yao Y., Xu Y., Li L., Gong Y., Zhang K., Zhang M., Guan Y., Chang L., Xia X. (2021). Pan-cancer circulating tumor DNA detection in over 10,000 Chinese patients. Nat. Commun..

[81] Hayashi T., Yoshida Y., Yamada T., Tanaka K., Shimaoka H., Kajitani R., Munechika T., Nagano H., Matsumoto Y., Komono A. (2022). Relationship between perioperative oncological evaluation and recurrence using circulating tumor DNA with KRAS mutation in patients with colorectal cancer. Cancer Med..

[82] Rakhit C.P., Trigg R.M., Le Quesne J., Kelly M., Shaw J.A., Pritchard C., Miguel Martins L. (2019). Early detection of pre-malignant lesions in a KRAS G12D -driven mouse lung cancer model by monitoring circulating free DNA. DMM Dis. Model. Mech..

[83] Skoulidis F., Heymach J.V. (2019). Co-occurring genomic alterations in non-small-cell lung cancer biology and therapy. Nat. Rev. Cancer.

[84] Chuang J.C., Neal J.W. (2015). Crizotinib as first line therapy for advanced ALK-positive non-small cell lung cancers. Transl. Lung Cancer Res..

[85] Shaw A.T., Yeap B.Y., Mino-Kenudson M., Digumarthy S.R., Costa D.B., Heist R.S., Solomon B., Stubbs H., Admane S., McDermott U. (2009). Clinical features and outcome of patients with non-small-cell lung cancer who harbor EML4-ALK. J. Clin. Oncol..

[86] Dagogo-Jack I., Ritterhouse L.L. (2020). The role of plasma genotyping in ALK- and ROS1-rearranged lung cancer. Transl. Lung cancer Res..

[87] Horn L., Whisenant J.G., Wakelee H., Reckamp K.L., Qiao H., Leal T.A., Du L., Hernandez J., Huang V., Blumenschein G.R. (2019). Monitoring Therapeutic Response and Resistance: Analysis of Circulating Tumor DNA in Patients With ALK+ Lung Cancer. J. Thorac. Oncol..

[88] Chow L.Q.M., Barlesi F., Bertino E.M., van den Bent M.J., Wakelee H.A., Wen P.Y., Chiu C.-H., Orlov S., Chiari R., Majem M. (2022). ASCEND-7: Efficacy and Safety of Ceritinib Treatment in Patients With ALK -Positive Non-Small Cell Lung Cancer Metastatic to the Brain and/or Leptomeninges. Clin. Cancer Res..

[89] Lin Y.T., Chiang C.L., Hung J.Y., Lee M.H., Su W.C., Wu S.Y., Wei Y.F., Lee K.Y., Tseng Y.H., Su J. (2021). Resistance profiles of anaplastic lymphoma kinase tyrosine kinase inhibitors in advanced non-small-cell lung cancer: A multicenter study using targeted next-generation sequencing. Eur. J. Cancer.

[90] Pailler E., Faugeroux V., Oulhen M., Mezquita L., Laporte M., Honore A., Lecluse Y., Queffelec P., NgoCamus M., Nicotra C. (2019). Acquired Resistance Mutations to ALK Inhibitors Identified by Single Circulating Tumor Cell Sequencing in ALK-Rearranged Non-Small-Cell Lung Cancer. Clin. Cancer Res..

[91] Oulhen M., Pawlikowska P., Tayoun T., Garonzi M., Buson G., Forcato C., Manaresi N., Aberlenc A., Mezquita L., Lecluse Y. (2021). Circulating tumor cell copy-number heterogeneity in ALK-rearranged non-small-cell lung cancer resistant to ALK inhibitors. npj Precis. Oncol..

[92] Sánchez-Herrero E., Serna-Blasco R., Ivanchuk V., García-Campelo R., Dómine Gómez M., Sánchez J.M., Massutí B., Reguart N., Camps C., Sanz-Moreno S. (2021). NGS-based liquid biopsy profiling identifies mechanisms of resistance to ALK inhibitors: A step toward personalized NSCLC treatment. Mol. Oncol..

[93] Hua G., Zhang X., Zhang M., Wang Q., Chen X., Yu R., Bao H., Liu J., Wu X., Shao Y. (2022). Real-world circulating tumor DNA analysis depicts resistance mechanism and clonal evolution in ALK inhibitor-treated lung adenocarcinoma patients. ESMO Open.

[94] Swalduz A., Ortiz-Cuaran S., Avrillon V., Marteau S., Martinez S., Clapisson G., Montane L., Pérol D., Green E., Howarth K. (2018). Fusion detection and longitudinal circulating tumor DNA (ctDNA) profiling in ALK+ non-small cell lung cancer (NSCLC) patients. J. Clin. Oncol..

[95] Takeuchi K., Soda M., Togashi Y., Suzuki R., Sakata S., Hatano S., Asaka R., Hamanaka W., Ninomiya H., Uehara H. (2012). RET, ROS1 and ALK fusions in lung cancer. Nat. Med..

[96] Lin J.J., Shaw A.T. (2017). Recent Advances in Targeting ROS1 in Lung Cancer. J. Thorac. Oncol..

[97] Dagogo-Jack I., Rooney M., Nagy R.J., Lin J.J., Chin E., Ferris L.A., Ackil J., Lennerz J.K., Lanman R.B., Gainor J.F. (2019). Molecular Analysis of Plasma From Patients With ROS1-Positive NSCLC. J. Thorac. Oncol..

[98] Bruno R., Fontanini G. (2020). Next Generation Sequencing for Gene Fusion Analysis in Lung Cancer: A Literature Review. Diagnostics.

[99] Mellert H.S., Alexander K.E., Jackson L.P., Pestano G.A. (2018). A Blood-based Test for the Detection of ROS1 and RET Fusion Transcripts from Circulating Ribonucleic Acid Using Digital Polymerase Chain Reaction. JoVE.

[100] Shaw A.T., Ou S.-H.I., Bang Y.-J., Camidge D.R., Solomon B.J., Salgia R., Riely G.J., Varella-Garcia M., Shapiro G.I., Costa D.B. (2014). Crizotinib in ROS1 -Rearranged Non–Small-Cell Lung Cancer. N. Engl. J. Med..

[101] Cho B.C., Lim S.M., Kim H.R., Lee J.S., Lee K.H., Lee Y.G., Min Y.J., Cho E.K., Lee S.S., Kim B.S. (2017). Open-label, multicenter, phase II Study of ceritinib in patients with non–small-cell lung cancer harboring ROS1 rearrangement. J. Clin. Oncol..

[102] Shaw A.T., Felip E., Bauer T.M., Besse B., Navarro A., Postel-Vinay S., Gainor J.F., Johnson M., Dietrich J., James L.P. (2017). Lorlatinib in non-small-cell lung cancer with ALK or ROS1 rearrangement: An international, multicentre, open-label, single-arm first-in-man phase 1 trial. Lancet. Oncol..

[103] Drilon A., Siena S., Ou S.H.I., Patel M., Ahn M.J., Lee J., Bauer T.M., Farago A.F., Wheler J.J., Liu S.V. (2017). Safety and Antitumor Activity of the Multitargeted Pan-TRK, ROS1, and ALK Inhibitor Entrectinib: Combined Results from Two Phase I Trials (ALKA-372-001 and STARTRK-1). Cancer Discov..

[104] Aldea M., Hendriks L., Mezquita L., Jovelet C., Planchard D., Auclin E., Remon J., Howarth K., Benitez J.C., Gazzah A. (2020). Circulating Tumor DNA Analysis for Patients with Oncogene-Addicted NSCLC With Isolated Central Nervous System Progression. J. Thorac. Oncol..

[105] Planchard D., Kim T.M., Mazieres J., Quoix E., Riely G., Barlesi F., Souquet P.J., Smit E.F., Groen H.J.M., Kelly R.J. (2016). Dabrafenib in patients with BRAFV600E-positive advanced non-small-cell lung cancer: A single-arm, multicentre, open-label, phase 2 trial. Lancet Oncol..

[106] Gautschi O., Milia J., Cabarrou B., Bluthgen M.V., Besse B., Smit E.F., Wolf J., Peters S., Früh M., Koeberle D. (2015). Targeted Therapy for Patients with BRAF-Mutant Lung Cancer: Results from the European EURAF Cohort. J. Thorac. Oncol..

[107] Facchinetti F., Lacroix L., Mezquita L., Scoazec J.Y., Loriot Y., Tselikas L., Gazzah A., Rouleau E., Adam J., Michiels S. (2020). Molecular mechanisms of resistance to BRAF and MEK inhibitors in BRAFV600E non–small cell lung cancer. Eur. J. Cancer.

[108] Jiang J., Gao J., Wang G., Lv J., Chen W., Ben J., Wang R. (2021). Case Report: Vemurafenib Treatment in Brain Metastases of BRAFS365L-Mutant Lung Papillary Cancer by Genetic Sequencing of Cerebrospinal Fluid Circulating Tumor DNA Detection. Front. Oncol..

[109] Li M., Zhang X. (2021). BRAF Mutations and Resistance of Non-Small Cell Lung Cancer to BRAF-Targeted Therapies Using Liquid Biopsy. Asia-Pac. J. Oncol. Nurs..

[110] Schreuer M., Meersseman G., Van Den Herrewegen S., Jansen Y., Chevolet I., Bott A., Wilgenhof S., Seremet T., Jacobs B., Buyl R. (2016). Quantitative assessment of BRAF V600 mutant circulating cell-free tumor DNA as a tool for therapeutic monitoring in metastatic melanoma patients treated with BRAF/MEK inhibitors. J. Transl. Med..

[111] Gautschi O., Milia J., Filleron T., Wolf J., Carbone D.P., Owen D., Camidge R., Narayanan V., Doebele R.C., Besse B. (2017). Targeting RET in Patients With RET-Rearranged Lung Cancers: Results From the Global, Multicenter RET Registry. J. Clin. Oncol..

[112] Ferrara R., Auger N., Auclin E., Besse B. (2018). Clinical and Translational Implications of RET Rearrangements in Non–Small Cell Lung Cancer. J. Thorac. Oncol..

[113] Gainor J.F., Curigliano G., Kim D.W., Lee D.H., Besse B., Baik C.S., Doebele R.C., Cassier P.A., Lopes G., Tan D.S.W. (2021). Pralsetinib for RET fusion-positive non-small-cell lung cancer (ARROW): A multi-cohort, open-label, phase 1/2 study. Lancet. Oncol..

[114] Drilon A., Oxnard G.R., Tan D.S.W., Loong H.H.F., Johnson M., Gainor J., McCoach C.E., Gautschi O., Besse B., Cho B.C. (2020). Efficacy of Selpercatinib in RET Fusion-Positive Non-Small-Cell Lung Cancer. N. Engl. J. Med..

[115] Rich T.A., Reckamp K.L., Chae Y.K., Doebele R.C., Iams W.T., Oh M., Raymond V.M., Lanman R.B., Riess J.W., Stinchcombe T.E. (2019). Analysis of cell-free DNA from 32,989 advanced cancers reveals novel co-occurring activating RET alterations and oncogenic signaling pathway aberrations. Clin. Cancer Res..

[116] Yeung V., Kim C., Kiedrowski L.A., Liu S.V., Reuss J.E. (2022). Use of on-therapy ctDNA monitoring in a patient with KIF5B-RET fusion positive advanced non-small cell lung cancer: A case report. Transl. Lung Cancer Res..

[117] Mo H., Liu P. (2017). Targeting MET in cancer therapy. Chronic Dis. Transl. Med..

[118] Awad M.M., Oxnard G.R., Jackman D.M., Savukoski D.O., Hall D., Shivdasani P., Heng J.C., Dahlberg S.E., Jänne P.A., Verma S. (2016). MET Exon 14 Mutations in Non-Small-Cell Lung Cancer Are Associated With Advanced Age and Stage-Dependent MET Genomic Amplification and c-Met Overexpression. J. Clin. Oncol..

[119] Ma P.C., Jagadeeswaran R., Jagadeesh S., Tretiakova M.S., Nallasura V., Fox E.A., Hansen M., Schaefer E., Naoki K., Lader A. (2005). Functional expression and mutations of c-Met and its therapeutic inhibition with SU11274 and small interfering RNA in non-small cell lung cancer. Cancer Res..

[120] Wolf J., Seto T., Han J.-Y., Reguart N., Garon E.B., Groen H.J.M., Tan D.S.W., Hida T., de Jonge M., Orlov S.V. (2020). Capmatinib in MET Exon 14-Mutated or MET-Amplified Non-Small-Cell Lung Cancer. N. Engl. J. Med..

[121] Camidge D.R., Otterson G.A., Clark J.W., Ignatius Ou S.H., Weiss J., Ades S., Shapiro G.I., Socinski M.A., Murphy D.A., Conte U. (2021). Crizotinib in Patients With MET-Amplified NSCLC. J. Thorac. Oncol..

[122] Le X., Paz-Ares L.G., Van Meerbeeck J., Viteri S., Galvez C.C., Baz D.V., Kim Y.-C., Kang J.-H., Schumacher K.-M., Karachaliou N. (2021). Tepotinib in patients (pts) with advanced non-small cell lung cancer (NSCLC) with MET amplification (METamp). J. Clin. Oncol..

[123] Xu J., Qu S., Sun N., Zhang W., Zhang J., Song Q., Lin M., Gao W., Zheng Q., Han M. (2021). Construction of a reference material panel for detecting KRAS/ NRAS/ EGFR/ BRAF/ MET mutations in plasma ctDNA. J. Clin. Pathol..

[124] Schrock A.B., Welsh A., Chung J.H., Pavlick D., Bernicker E.H., Creelan B.C., Forcier B., Ross J.S., Stephens P.J., Ali S.M. (2019). Hybrid Capture–Based Genomic Profiling of Circulating Tumor DNA from Patients with Advanced Non–Small Cell Lung Cancer. J. Thorac. Oncol..

[125] Tie J., Cohen J., Lahouel K., Lo S.N., Wang Y., Wong R., Shapiro J.D., Harris S.J., Khattak M.A., Burge M.E. (2022). Adjuvant chemotherapy guided by circulating tumor DNA analysis in stage II colon cancer: The randomized DYNAMIC trial. J. Clin. Oncol..

